# A Comprehensive Evaluation of the Predictive Abilities of Fetal Electrocardiogram-Derived Parameters during Labor in Newborn Acidemia: Our Institutional Experience

**DOI:** 10.1155/2018/3478925

**Published:** 2018-05-17

**Authors:** Ning Tian, Weiyuan Zhang

**Affiliations:** Obstetrical Department, Beijing Obstetrics and Gynecology Hospital, Capital Medical University, Beijing 100026, China

## Abstract

This study aimed to identify cardiotocography patterns that discriminate fetal acidemia newborns by comprehensively evaluating the parameters obtained from Holter monitoring during delivery. Between June 1, 2015, and August 1, 2016, a prospective observational study of 85 patients was conducted using fetal Holter monitoring at the Beijing Obstetrics and Gynecology Hospital, Capital Medical University, China. Umbilical cord blood was sampled immediately after delivery and fetal acidemia was defined as umbilical cord arterial blood pH < 7.20. Fetal electrocardiogram- (FECG-) derived parameters, including basal fetal heart rate (BFHR), short-term variation (STV), large acceleration (LA), deceleration capacity (DC), acceleration capacity (AC), proportion of episodes of high variation (PEHV), and proportion of episodes of low variation (PELV), were compared between 16 fetuses with acidemia and 47 without. The areas under the curve (AUC) of receiver operating characteristics (ROC) were calculated. Although all the computerized parameters showed predictive values for acidemia (all AUC > 0.50), STV (AUC = 0.84, *P* < 0.001), DC (AUC = 0.84, *P* < 0.001), AC (AUC = 0.80, *P* < 0.001), and PELV (AUC = 0.71, *P* = 0.012) were more strongly associated with fetal acidemia. Our institutional experience suggests that FECG-derived parameters from Holter monitoring are beneficial in reducing the incidence of neonatal acidemia.

## 1. Introduction

Electronic fetal heart rate monitoring (EFM), first introduced at Yale University in 1958 [1], is the most commonly used approach in obstetrics to assess fetal well-being during labor [2–4]. EFM is mainly used to identify fetal hypoxia in an attempt to prevent subsequent perinatal asphyxia and acidemia [4, 5]. The association of fetal acidemia with seizures, cerebral palsy, multiorgan dysfunction, hypoxic ischemic encephalopathy, and long-term neurological deficits highlights the critical roles that EFM plays in the assessment of a neonate's risk of morbidity and death [6–11]. The primitive EFM is based on Doppler ultrasonography and has a low predictive value for intrauterine abnormalities of the fetus [3], possibly because of the variations in short monitoring duration (normally 20–40 min), interference from the maternal heart rate (MHR) and fetal sleeping cycle, and fetal or maternal movement, as well as the subjective assessments by medical staff.

Recently, a noninvasive fetal electrocardiogram (FECG) monitor, featuring continuous monitoring of the fetal heart rate (FHR) by measuring the electrical signals of the fetal heart during labor and delivery, has been approved for clinical use in many countries [12–14]. The feasibility and accuracy of long-term transabdominal FECG monitoring have been demonstrated by its variability as being well correlated with scalp electrode recordings [15]. FHR detection using abdominal FECG has proved to be more reliable and accurate than ultrasound because FECG has a higher ability to discriminate MHR [16–18]. It is now clear that intrapartum FHR determination using maternal skin-surface electrodes is superior in accuracy and reliability to monitoring that relies on Doppler ultrasound technology [16].

Despite its widespread use over many decades, conventional FHR analysis has important limitations regarding its ability to predict acidemia. Expert assessments have poor reproducibility and the method has only moderate specificity, leading to potentially unnecessary interventions [19–21]. Although an international standardization of FHR interpretations has been proposed [4, 22], the interpretations are associated with considerable inter- and intraobserver differences [23, 24]. This is why multiple computational methods for FHR analysis have been proposed, all aimed at providing more reproducible and physiologically relevant evaluations [25]. Nevertheless, only a limited number of such methods have been tested during labor [2, 3, 25]. It is imperative to search for effective physiology-based approaches for more accurate deciphering of FHR data.

Holter monitoring based on FECG technology has been introduced in China and is being tested in clinics. The introduction of FECG into clinical practice preceded the studies on the pathophysiological interpretation of the parameters assessed, and guidelines were based on the opinion of experts rather than on sound evidence [4, 22]. Because variable and inconsistent interpretation of FHR tracings of FECG might affect management decisions regarding fetal acidemia, a systematic approach to interpreting the patterns is important. Toward this goal, we performed a comprehensive evaluation of seven parameters that were derived from Holter monitoring data of parturients in our hospital. These parameters are highly associated with the integrative capacity of the fetal nervous system. We are hopeful that our institutional experience would help better discriminate between acidemia and nonacidemia fetuses during labor.

## 2. Materials and Methods

### 2.1. Ethics Considerations

Ethical clearance and approval were obtained from the Institutional Center Ethics Review Committee at Beijing Maternity Hospital, Capital University of Medical Sciences, China. The study was conducted in accordance with the Declaration of Helsinki and the 11th decree of the People's Republic of China National Health and Family Planning Commission, effective since December 1, 2016. The study protocol was carefully explained to the participants and participation was fully voluntary. Written informed consent was obtained from all participants and they agreed on the publication of their individual data. The attending obstetricians were blind to the information obtained from computerized FHR analyses, which could not be used for patient management.

### 2.2. Study Design and Participants

A prospective study was performed using a cohort of pregnant women who delivered at the Beijing Obstetrics and Gynecology Hospital, Capital Medical University, between June 1, 2015, and August 1, 2016. The inclusion criteria were (1) singleton, (2) vertex-presenting fetuses, (3) 37–41 weeks of gestation, and (4) with Holter monitoring during the entire delivery. All patients had at least one continuous electronic FHR monitoring of >14.0 h within 1.0 week of delivery. The analyses were conducted using only the data from patients with complete information in the clinical records and adequate FHR monitoring quality (recording quality ≥ 60.0%). Patients were excluded if they (1) had evidence of fetal structural anomalies, (2) had complications from infection or maternal drug exposure, or (3) did not have an umbilical arterial blood (UAB) gas measurement.

Demographic and clinical information (maternal age, gestational age, complications, body mass index (BMI), labor type, mode of delivery, and neonatal outcome) was extracted from the medical records. Patients were divided into a case group with fetal metabolic acidemia at birth (acidemia group) and a control group with normal umbilical cord gases (nonacidemia group). Umbilical cord blood was sampled immediately after delivery for subsequent measurement of arterial cord blood gases using a STAT300 blood gas analyzer (Abbott Laboratories, Chicago, IL, USA). Fetal acidemia was defined as umbilical cord arterial pH < 7.20, as previously reported [26–28]. The fetuses in the acidemia group were compared with those in the nonacidemia group. The individual FHR characteristics and their differences and correlations were assessed in relation to the newborn UAB pH.

### 2.3. FECG-Based Holter Monitoring

**Figure 1:**
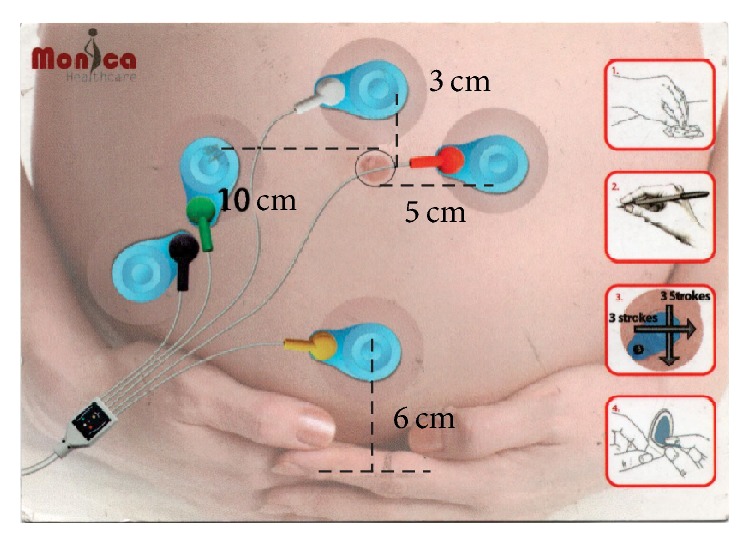
Representative image showing the arrangement of the five cutaneous electrodes on the mother's abdomen during Holter monitoring and the abdominal fetal electrocardiogram device next to the parturient.

FECG monitoring was performed by researchers who thoroughly explained the purpose and noninvasive methods. FHR tracings were archived electronically by an FECG-based Monica AN24 Holter monitor (Monica Healthcare Ltd., Nottingham, UK). The Monica AN24 device uses five Blue Sensor VLC-00S ECG electrodes (Ambu Ltd., St. Ives, UK) to record electrophysiological signals from the parturient's abdominal wall in a standardized manner (Figure 1). The data obtained by the FECG monitor were transmitted wirelessly to a bedside personal computer and stored for analysis. The FHR values from the device were updated every 0.25 sec. All FHR data were recorded digitally, and all data could be printed on the spot or on demand later, at a printing speed of 1.0 cm/min. The FHR, MHR, and uterine contraction data were extracted for subsequent analysis. The methods used for FECG signal extraction and analysis were described in detail by Piéri et al. [29]. In this study, fetal heartbeat recording quality (RQ, %) was specifically defined as the percentage of the effective fetal heartbeat data among the total fetal heartbeat data. Namely, it was expressed as RQ = 100%-fetal heartbeat data loss% [30–32]. The device is supposed to collect 4 points within 1 sec, hence 240 points within 1 min. For example, if 240 points are all collected within 1 min, QR is 100%; if only 144 points were collected, it is 60%.

### 2.4. Parameters Used for Comparison

The valid electronically monitored data derived from eligible patients based on the inclusion and exclusion criteria were subjected to further in-depth analyses. Seven parameters derived from Holter monitoring were used for comparison between the acidemia and nonacidemia groups. While the basal FHR (BFHR), short-term variation (STV), large acceleration (LA), proportion of episodes of high variation (PEHV), and proportion of episodes of low variation (PELV) were extracted from Monica AN24 coupled with the Monica VS software, the deceleration capacity (DC) and acceleration capacity (AC) were calculated by the software developed by the Laboratory of Bioelectronics and Medical Applications, Beijing University of Technology, China. The various measurements produced by built-in software are defined below.

BFHR refers to the mean value of the FHR for >10.0 min without the effects of fetal movement and contractions. The normal range is 110.0–160.0 pbm [33].

STV is the variation difference between each heartbeat, which is not visible to the naked eye and can only be obtained by a computer or FECG [34–36]. Monica AN24 automatically extracts four R-R interphase values per second, and the average STV value is calculated in real time. STV thus refers to the change of FHR at individual heartbeat, namely, the divergence of each FHR of one beat from the next. This variation estimates the interval between two systoles, expressed by ms [37–40]. The STV is computed from any minute of recording that does not contain deceleration or part of deceleration and does not have a high signal loss (more than 10 s of loss). For each valid minute, the STV is computed as the average of the difference of the adjacent 3.75 s periods of FHR [37–39]. STV is calculated using the following formula:

(1) $$\begin{array}{c} STV=\frac{1}{16M}\overset{16M}{\underset{i=1}{\sum }}\left|\;sm\left(\;i+1\;\right)-sm\left(\;i\;\right)\;\right|, \end{array}$$

where *M* is the number of minutes of the signal and *sm*(*i*) are the values of *x*(*i*) on each period of 3.75 s [41].

DC is a marker of autonomic function that indicates the modulating ability by the vagus nerve of a faster cardiac cycle during heart rate adjustment. DC is calculated using phase-rectified signal averaging (PRSA) [42] using the following formula:

(2) $$\begin{array}{c} DC=\left[\;X\left(\;0\;\right)+X\left(\;1\;\right)-X\left(\;-1\;\right)-X\left(\;-2\;\right)\;\right]\times \frac{1}{4}, \end{array}$$

where *X* indicates the mean value of deceleration points during the cardiac cycle. DC is denoted as a positive value in milliseconds. *X* is the coordinate value of each cycle, including the center, right 1, left 1, and left 2. The average is the mean value of the four cycles. The selected cycles are fixed [42].

AC is a marker of autonomic function that indicates the modulating ability by the sympathetic nervous system of the slower cardiac cycle during heart rate adjustment. AC is calculated by PRSA [42] using the following formula:

(3) $$\begin{array}{c} AC=\left[\;X\left(\;0\;\right)+X\left(\;1\;\right)-X\left(\;-1\;\right)-X\left(\;-2\;\right)\;\right]\times \frac{1}{4}, \end{array}$$

where *X* indicates the mean value of deceleration points during the cardiac cycle. AC is denoted by a negative value in milliseconds. In this study, the absolute value of AC was used as the study parameter.

LA is classified as an increase of greater than 15 pbm and lasting for more than 15 seconds. LA is defined as a good sign of fetal wellness [43]. During fetal development, acceleration starts at 25-26 weeks of pregnancy, and the improvement of the action mechanism takes place after 28-29 weeks. Therefore, acceleration is a specific physiological phenomenon of late pregnancy. There are mainly two types of acceleration: aperiodic acceleration, also named sporadic acceleration, which occurs upon stimulation of fetal movement, pelvic examination, or abdominal palpation, and periodic acceleration, which refers to the one accompanying uterine contraction, in which FHR rises and uterine contraction occurs synchronously.

**Figure 2:**
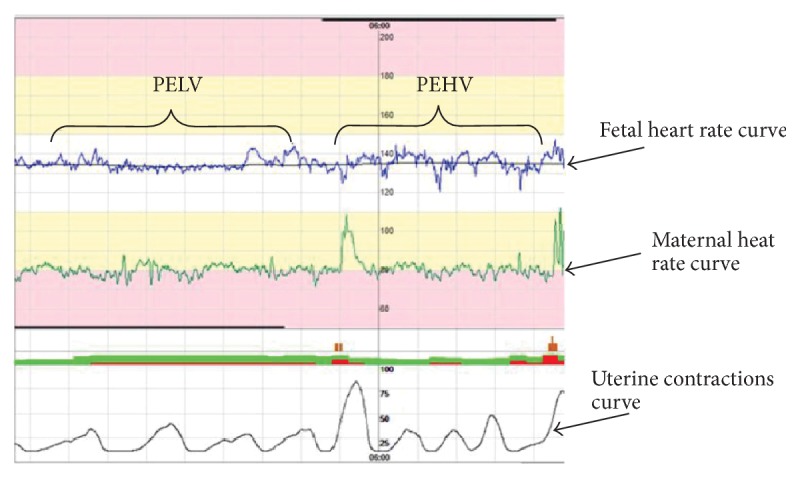
Representative analyses for deciphering the episodes of high and low variations during fetal electrocardiogram- (FECG-) based Holter monitoring.* Notes*. PEHV: proportion of episodes of high variation; PELV: proportion of episodes of low variation.

PEHV occurs from ~28.0 weeks' gestation onward. A healthy fetus cycles between episodes of active and quiet sleep. Active sleep is associated with acceleration, high FHR variation, and clusters of fetal movements, whereas quiet sleep is associated with low FHR variation and reduced fetal movement (Figure 2). Episodes of HV refer to the period in which the fetal cardiac cycle interval is ≥32.0 ms for each minute of a minimum of consecutive 5.0 min intervals. The ratio of EHV to the entire valid monitoring time is defined as PEHV [32, 44].

PELV refers to the period in which the fetal cardiac cycle interval is ≤30.0 ms for each minute of a minimum of consecutive 5.0 min intervals. The ratio of ELV to the entire valid monitoring time is defined as PELV [32, 44].

### 2.5. Statistical Analysis

Statistical analyses were performed using SPSS 19.0 (IBVM, Armonk, NY, USA). All continuous variables were checked for normal distribution by the Kolmogorov–Smirnov normality test. Normally distributed variables are expressed as mean ± standard deviation, while skewed variables are expressed as the median (range). The unpaired two-tailed Student *t*-tests and Kolmogorov–Smirnov tests were applied to examine the difference between the two groups for normally and non-normally distributed parameters, respectively. Categorical data were analyzed using the chi-squared test: Pearson's chi-square was used when* T* > 5 and* N* > 40, the continuity correction was used when* T* ≥ 1 and* N* > 40, and Fisher's exact test was used when* T* < 1. The area under the curve (AUC) of the receiver operating characteristic (ROC) was computed as a measure of the ability of FHR parameters to discriminate between acidemia and nonacidemia fetuses. *P* < 0.05 was considered statistically significant.

## 3. Results

### 3.1. Patient Enrollment and Grouping

Eighty-five pregnant women were enrolled in this study. Based on our inclusion and exclusion criteria, 63 of these were finally included in the analysis. Eight patients failed the FECG Holter monitoring because of operational error or incomplete data recording. Another 13 patients were excluded because of poor FECG data quality or loss, and one patient did not have a UAB gas measurement. The study population consisted of 63 patients who were divided into two groups based on the newborn UAB pH values as follows: acidemia group (pH < 7.20, *n* = 16) and nonacidemia (control) group (pH ≥ 7.20, *n* = 47).

### 3.2. Demographic and Obstetric Characteristics of the Study Population

As shown in Table 1, the acidemia group displayed UAB pH values ranging from 6.98 to 7.19 with a mean value of 7.13, while the nonacidemia group had UAB pH values ranging from 7.21 to 7.38 with a mean value of 7.26. In addition to the UAB pH values, the percentage of newborns admitted to the neonatal intensive care unit (NICU) was significantly different between the acidemia and nonacidemia groups (18.8 versus 2.1%, resp.; *P* = 0.02).

With respect to parturient characteristics, the mothers of both groups were of similar age (30.8 ± 2.3 years for the acidemia group; 29.8 ± 3.0 years for the nonacidemia group) and of similar gestational age at delivery (39.6 ± 0.9 versus 39.4 ± 1.2 weeks, resp.) and had similar BMIs (21.8 ± 4.5 versus 21.4 ± 3.6 kg/m^2^, resp.). The vast majority of patients in both groups were nulliparous (100% versus 95.7%, resp.). None of the patients had a history of C-section. Only two mothers in the nonacidemia group had a history of vaginal delivery. Moreover, similar incidences of gestation-associated complications, including gestational diabetes mellitus, premature rupture of the membrane, gestational hypertension, or preeclampsia, were observed in the two groups. No significant differences in the mode of fetal delivery (*P* = 0.40 for vaginal; *P* = 0.15 for operative vaginal; *P* = 0.40 for C-section) were identified (Table 1).

With respect to fetal characteristics, both groups of babies had comparable (all *P* > 0.05) body weights at delivery (3598 ± 415 g for the acidemia group and 3464 ± 463 g for the control group) and comparable labor times (550 ± 241 versus 530 ± 290 min, resp.). All babies exhibited superior healthy newborn conditions, as evidenced by Apgar scores ≥7 for all of them. Taken together, except for the UAB pH values and NICU admission rate, no significant differences were observed in any of the other maternal or fetal characteristics (Table 1).

### 3.3. Comparisons of FECG Parameters between the Acidemia and Nonacidemia Groups

As previously reported [27], our results also demonstrated that variable deceleration and late deceleration were significantly more frequent in the acidemia group than in the nonacidemia group during labor. The following variables were found to be significantly different between the acidemia and nonacidemia groups: STV (5.91 ± 1.17 versus 8.46 ± 2.05 ms, resp.; *P* = 0.03), DC (1.69 ± 0.38 versus 2.42 ± 0.64 ms, resp.; *P* = 0.04), AC (1.67 ± 0.39 versus 2.31 ± 0.66 ms, resp.; *P* = 0.03), and PELV (17.45 ± 7.86 versus 11.26 ± 5.46%, resp.; *P* = 0.04); nevertheless, no significant differences were observed between the acidemia and nonacidemia groups for BFHR (140.67 ± 8.89 versus 136.67 ± 8.34 pbm, resp.; *P* = 0.52), LA (3.91 ± 2.56 versus 6.64 ± 3.58 times/h, resp.; *P* = 0.24), and PEHV (36.07 ± 11.49 versus 42.41 ± 13.75%, resp.; *P* = 0.72). Thus, STV, DC, AC, and PELV were significantly associated with acidemia (Table 2).

### 3.4. Predictive Values of FECG Parameters for Acidemia

**Figure 3:**
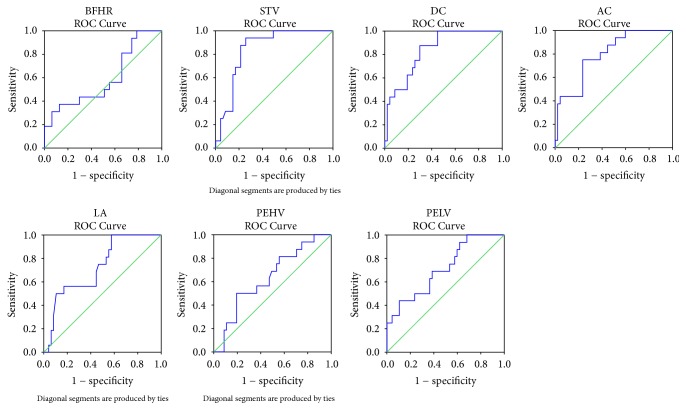
Receiver operating characteristic (ROC) curves showing the predictive value of each computerized fetal electrocardiogram- (FECG-) derived fetal heart rate (FHR) parameter and acidemia at birth. Basal fetal heart fate (BFHR), AUC = 0.59, *P* = 0.276. Short-term variation (STV), AUC = 0.84, *P* < 0.001. Deceleration capacity (DC), AUC = 0.84, *P* < 0.001. Acceleration capacity (AC), AUC = 0.80, *P* < 0.001. Large acceleration (LA), AUC = 0.73, *P* = 0.240. Proportion of episodes of high variation (PEHV), AUC = 0.64, *P* = 0.077. Proportion of episodes of low variation (PELV), AUC = 0.71, *P* = 0.012. AUC: area under the curve.

ROC curves were constructed to analyze the correlation between each computerized FECG-derived FHR parameter and the threshold of acidemia at birth. Variations in the sensitivity and specificity of these parameters for screening neonatal acidemia are presented as ROC curves in Figure 3. AUC represents the overall performance of the test. AUC of ~1 indicates excellent diagnostic ability, while AUC ~0.50 reflects no diagnostic ability. As shown in Table 3, STV (AUC, 0.84; 95% CI, 0.75–0.94; *P* < 0.001), DC (AUC, 0.84; 95% CI, 0.74–0.94; *P* < 0.001), AC (AUC, 0.80; 95% CI, 0.68–0.91; *P* < 0.001), and PELV (AUC, 0.71; 95% CI, 0.57–0.86; *P* = 0.012) apparently show predictive abilities for discriminating neonatal acidemia, as demonstrated by their AUC values that significantly differed from the value of 0.50. It is evident that STV, DC, and AC are superior to PELV as indicators of fetal acidemia because all of them had Youden's index values higher than that of PELV (Table 3). BFHR, LA, and PEHV also displayed AUC values >0.50, but they were much less predictive because all the *P* values were >0.05 and large variations were evident on the ROC curves for these parameters (Table 3, Figure 3).

## 4. Discussion

### 4.1. Principle Findings

Fetal acidemia is a major cause of neonatal morbidity and mortality resulting from an acute or progressive imbalance between an inadequate oxygen supply and increased fetal metabolic demand [6]. More new and adjunctive methods are being investigated to make FHR tracing more readily discernable for discriminating between a fetus with metabolic acidemia and a healthy fetus [3, 45, 46]. Our case-control study involving 16 parturients with acidemia fetuses and 47 parturients with normal fetuses suggests that parameters such as STV, DC, and AC are highly predictive of an adverse neonatal outcome in terms of metabolic acidemia.

### 4.2. Meanings of the Findings

Based on FECG technology, the Holter monitoring system automatically calculates FHR variability using a computer-aided fetal monitoring system and ensures the highest possible accuracy of heart interval measurements [13, 14] and more advanced parameters [16–18] and superior sensitivity and accuracy that are comparable to intrauterine monitoring [47]. BFHR and STV have been used in a clinical study on fetal heart rate changes since the 1960s [48]. STV represents the transient changes of FHR and is superior to long-term FHR variation in terms of predicting fetal hypoxia, acidemia, and even intrapartum death [35]. By first defining STV as the 1/16-minute period-period variation, Street et al. [35] identified that STV provided better detection of preterminal records as judged by metabolic acidemia at delivery or intrauterine death. Because fetal compromise was found on occasion to be associated with a slow sinusoidal FHR rhythm, the decline in STV value can significantly increase the risk of fetal distress and acidemia, as previously reported [30]. Williams and Galerneau [49] also concluded that the most significant intrapartum FHR parameter to predict the development of significant acidemia is the presence of minimal/absent variability for at least 1.0 h as a solitary abnormal finding or in conjunction with late deceleration in the absence of acceleration. Nevertheless, Aernout et al. [28] found that the performance of STV for predicting neonatal acidemia was poor in women with preeclampsia.

Our results indicate that STV, DC, and AC from the acidemia group were significantly lower than those in the nonacidemia group. A number of different mechanisms may cause acidemia in a fetus and the present study was not designed to determine the exact cause of acidemia. Nevertheless, the different causes of acidemia may include autonomic system dysregulation such as vagal excitability (which often leads to fetal distress and neonatal asphyxia), any disruption of the adrenergic system versus cholinergic system balance, fetal movement, fetal age, and gestational complications that affect placenta/fetus blood and gas exchange (such as preeclampsia, thrombotic diseases, gestational diabetes, oligohydramnios, abnormal torsion and twining of the umbilical cord, abnormal placental position, or placental abruption), eventually resulting in hypoxemia, hypercapnia, and metabolic acidosis [38, 50–55]. A recent study using an* in vivo *sheep model also found that increasing values of AC/DC suggested that activation of the fetal autonomic nervous system as the time evolution of AC/DC correlated well with the acid-base balance [52].

It was concluded that fetal movements could be verified by the existence of large acceleration (LA) on the FHR tracing data [56]. LA was associated with 78.6% of fetal movements felt by the mother and 99.6% of fetal movements seen by real-time ultrasonography [56]. The acidemia group had an obvious but not significantly lower mean LA value (3.91 times/h) compared with the nonacidemia group (6.64 times/h) (Table 2), which also reflects a lower tension of the fetal autonomic nervous system resulting from acidemia. PEHV and PELV display the distribution of high-variation and low-variation FHR in continuous FECG monitoring, which correspond to the fetal active sleep cycle and quiet sleep cycle, respectively [32, 44]. Analysis of a large archive of traces from healthy fetuses by Serra et al. [57] indicated that FHR acceleration, short- and long-term variation overall, duration of episodes of high and low variation, and variations in high episodes increased with advancing gestation. Dawes et al. [31] found that the PELV values in healthy fetuses at gestation ages of 28–40 weeks were lower than in fetuses with chronic hypoxemia and metabolic acidemia, although all fetuses had comparable STV values. Nevertheless, the present study suggests that a higher PELV at delivery is associated with a higher incidence of fetal acidemia (Table 2, Figure 1). Therefore, additional studies using a larger sample size are warranted to further decipher the correlation between PELV and fetal metabolic acidemia. Taken together, the clinical application of maternal-fetal Holter monitoring based on the FECG technique is still in the initial stages. The trends of changes for STV, DC, AC, PELV, and other quantitative parameters need further investigation, and their relationship with a poor neonatal outcome is one of the focuses in future clinical research.

### 4.3. Clinical and Research Implications

The obstetric community unanimously accepted the classification system proposed by the National Institute of Child and Human Development (NICHD) consensus panel [4]. The NICHD panel proposed a three-category system in which normal status and pathological status were well defined, but leaving a wide undetermined category that gathered >80.0% of FHR tracings, for which recommendations were not clear [5, 58]. The NICHD system was criticized by some investigators for its low validity and controversial utility [59–62]. Therefore, our investigations on the comprehensive evaluation of seven FECG-derived parameters substantially complemented the prevailing standards for better interpretation of EFM patterns. Our improved ability using DC, AC, STV, and PELV to predict acidemia over the existing taxonomy highlights the importance of the discovery of new ways to quantify and interpret the complex EFM patterns at bedside. The further validation of our institutional experience would definitely help minimize the possible adverse effects caused by complementary methods, such as fetal scalp-blood sampling, to diagnose fetal acidemia [63, 64].

The interpretation of FHR patterns is reported to be associated with considerable inter- and intraobserver differences [23, 24]. As a result, the scientific value of FHR in clinical settings could be minimized by the ambiguity raised from conflicting interpretations of FHR patterns as well as the subsequent clinical actions [5, 62, 65]. Nevertheless, there is consensus that the expert and algorithm-assisted FHR interpretation has the potential to improve standard clinical performance by facilitating the early recognition of tracing that is associated with metabolic acidemia [66]. Recently, a large population-based study showed that centralization of FHR monitoring with the help of specialists to interpret the results and determine clinical actions achieved an effective decrease in the rate of fetal acidemia without an increase in the rate of C-section births [67]. This implies that our institutional experience in predicting acidemia through FECG-derived parameters can be incorporated into training the attending obstetricians to help them take action early enough to decrease the incidence of neonatal acidemia and intrapartum death. Collectively, our findings will assist clinicians as well as researchers in making more informed decisions about neonatal management.

### 4.4. Strengths and Weaknesses

The current investigation had several strengths and limitations. The strength of this study was the study design, which was a cohort of the Chinese population with UAB gas measurement data. It was prospective and blinded, and those analyzing the FCTG data were not aware of the fetal acidemia status at the time of data interpretation. The major limitation of the current study was its sample size of only 63 patients and the two groups had different numbers of patients but were still within the 1 : 4 ratio [68–70]. The patients included in this study were also drawn from a single institution and thus were subject to referral bias. Another limitation was that we do not have neonatal follow-up data or outcomes with which to correlate blood gas results. Nevertheless, the objective of the study was to establish the predictive abilities of the venous samples and establish cutoffs that would effectively rule out acidemia. In addition, we focused on the analysis of pH only, although both pH and base deficit are two parameters that are considered when determining pathologic acidemia [71–73]. Adjustments of defining acidemia based on both venous blood pH and base deficit as applicable to improve prediction accuracy deserve further evaluation. Furthermore, predictive models such as ours should undergo external validation. Thus, we plan to use another database to validate our current findings. Nevertheless, our intrapartum diagnosis of fetal acidemia cannot prevent birth acidemia related to chronic fetal hypoxia, in which damage might have already occurred before admission for delivery.

## 5. Conclusions

Our study shows that electronic FHR monitoring can provide clinicians with useful data for labor management, provided those data are interpreted using a strictly standardized system. Our institutional experience suggests that STV, DC, and AC are powerful predictors for hypoxic-acidemic insult in fetuses. It is our belief, based on the data presented, that broad and universal FECG-based Holter monitoring at the time of delivery would be a beneficial clinical practice to better stratify acidemia-associated neonatal risks.

## Figures and Tables

**Table 1:** Main maternal and fetal characteristics of the studied population in relation to the newborn umbilical artery blood pH (pH > 7.20, nonacidemia; pH ≤ 7.20, acidemia).

	Total *N* = 63 (%)	Acidemia *N* = 16 (%)	Nonacidemia *N* = 47 (%)	*P* value
Maternal age, y (mean ± SD)	30.08 ± 2.89	30.81 ± 2.34	29.83 ± 3.04	0.93
Maternal age ≥ 35 y^*∗∗*^	5 (7.9)	1 (6.3)	4 (8.5)	1.00
Gestational age at delivery, wk (median, range)	39.00 (37, 41)	39.00 (38, 41)	40.00 (37, 41)	0.77
BMI (median, range)	20.60 (15.8, 33.2)	20.70 (16.5, 31.7)	20.60 (15.8, 33.2)	0.91
BMI ≥ 30^*∗∗*^	4 (6.3)	2 (12.5)	2 (4.3)	0.57
Smoking^*∗∗*^	4 (6.3)	1 (6.3)	3 (6.4)	1.00
Any gestational hypertension or preeclampsia^*∗∗*^	6 (9.5)	2 (12.5)	4 (8.5)	1.00
Gestational DM^*∗∗*^	15 (23.8)	5 (31.3)	10 (21.3)	0.64
Premature rupture of membrane^*∗*^	20 (31.7)	4 (25.0)	16 (34.0)	0.50
Nulliparous^*∗*^	61 (96.8)	16 (100)	45 (95.7)	0.40
Prior C-section^*∗∗*^	0	0	0	-
Prior vaginal delivery^#^	2 (3.2)	0 (0)	2 (4.3)	0.55
Labor type				
Spontaneous^*∗*^	55 (87.3)	13 (81.2)	42 (89.4)	0.40
Induced^*∗∗*^	12 (19.0)	4 (25.0)	8 (17.0)	0.74
Prostaglandin^*∗∗*^	10 (15.9)	3 (18.8)	7 (14.9)	1.00
Birth weight, g (mean ± SD)	3498.17 ± 452.00	3598.13 ± 414.75	3464.15 ± 463.27	0.72
Birth weight > 4000 g^*∗∗*^	9 (14.3)	3 (18.8)	6 (12.8)	0.86
Birth weight < 2500 g	0	0	0	-
Mode of delivery				
Vaginal^*∗*^	55 (87.3)	13 (81.3)	42 (89.4)	0.40
Operative vaginal^*∗∗*^	6 (9.5)	3 (18.8)	3 (6.4)	0.34
Cesarean^*∗∗*^	8 (12.7)	3 (18.7)	5 (10.6)	0.68
Labor time, min (median, range)	495.00 (43, 1295)	491.00 (212, 1029)	495.00 (43, 1295)	0.31
Asphyxia (Apgar ≤ 7)	0	0	0	-
Arterial pH (median, range)	7.24 (6.98, 7.38)	7.16 (6.98, 7.19)	7.25 (7.21, 7.38)	<0.01
NICU admission^*∗∗*^	4 (6.3)	3 (18.8)	1 (2.1)	0.02

BMI: body mass index; DM: diabetes mellitus; NICU: neonatal intensive care unit. ^*∗*^Pearson's chi-square test. ^*∗∗*^Continuity correction. ^#^Fisher's exact test.

**Table 2:** Comparison of computerized FECG-derived FHR parameters in relation to acidemia.

	Total	Acidemia	Nonacidemia	*P* value
	*n* = 63	*n* = 16	*n* = 47	
BFHR, pbm	137.70 ± 8.60	140.67 ± 8.89	136.67 ± 8.34	0.52
STV, ms	7.81 ± 2.17	5.91 ± 1.17	8.46 ± 2.05	0.03
DC, ms	2.24 ± 0.67	1.69 ± 0.38	2.42 ± 0.64	0.04
|AC|, ms	2.15 ± 0.67	1.67 ± 0.39	2.31 ± 0.66	0.03
LA, times/h^*∗*^	5.94 ± 3.54	3.91 ± 2.56	6.64 ± 3.58	0.24
PEHV, %	40.80 ± 13.41	36.07 ± 11.49	42.41 ± 13.75	0.72
PELV, %	12.83 ± 6.67	17.45 ± 7.86	11.26 ± 5.46	0.04

FECG: fetal electrocardiogram; FHR: fetal heart rate; BFHR: basal fetal heart rate; STV: short-term variation; DC: deceleration capacity; AC: acceleration capacity; LA: large acceleration; PEHV: proportion of episodes of high variation; PELV: proportion of episodes of low variation. ^*∗*^Kolmogorov–Smirnov test.

**Table 3:** Variation in predictive values of computerized FECG-derived FHR parameters for neonatal acidemia by ROC curves analysis.

	AUC	Asymptotic 95% CI	*P* value	Sensitivity	Specificity	Youden's index
BFHR, pbm	0.59	0.42–0.76	0.276	0.31	0.64	0.25
STV, ms	0.84	0.75–0.94	<0.001	0.94	0.26	0.68
DC, ms	0.84	0.74–0.94	<0.001	0.88	0.7	0.58
|AC|, ms	0.80	0.68–0.91	<0.001	0.75	0.23	0.52
LA, times/h	0.73	0.59–0.86	0.240	1	0.57	0.43
PEHV, %	0.64	0.49–0.79	0.077	0.5	0.19	0.31
PELV, %	0.71	0.57–0.86	0.012	0.94	0.62	0.32

FECG: fetal electrocardiogram; FHR: fetal heart rate; CI: confidence interval; BFHR: basal fetal heart rate; STV: short-term variation; DC: deceleration capacity; AC: acceleration capacity; LA: large acceleration; PEHV: proportion of episodes of high variation; PELV: proportion of episodes of low variation; ROC: receiver operating characteristics.
